# Time Restricted Eating: A Dietary Strategy to Prevent and Treat Metabolic Disturbances

**DOI:** 10.3389/fendo.2021.683140

**Published:** 2021-08-12

**Authors:** Bettina Schuppelius, Beeke Peters, Agnieszka Ottawa, Olga Pivovarova-Ramich

**Affiliations:** ^1^Research Group Molecular Nutritional Medicine, Department of Molecular Toxicology, German Institute of Human Nutrition Potsdam-Rehbruecke, Nuthetal, Germany; ^2^Institute of Nutritional Science, University of Potsdam, Nuthetal, Germany; ^3^Institute of Human Nutrition and Food Science, Faculty of Agriculture and Food Sciences, Christian-Albrecht-University Kiel, Kiel, Germany; ^4^Charité – Universitätsmedizin Berlin, Corporate Member of Freie Universität Berlin, Humboldt-Universität zu Berlin, and Berlin Institute of Health, Berlin, Germany; ^5^German Center for Diabetes Research (DZD), München-Neuherberg, Germany

**Keywords:** time restricted eating, circadian clock, chrononutrition, glucose metabolism, lipid metabolism, metabolic diseases

## Abstract

Time-restricted eating (TRE), a dietary approach limiting the daily eating window, has attracted increasing attention in media and research. The eating behavior in our modern society is often characterized by prolonged and erratic daily eating patterns, which might be associated with increased risk of obesity, diabetes, and cardiovascular diseases. In contrast, recent evidence suggests that TRE might support weight loss, improve cardiometabolic health, and overall wellbeing, but the data are controversial. The present work reviews how TRE affects glucose and lipid metabolism based on clinical trials published until June 2021. A range of trials demonstrated that TRE intervention lowered fasting and postprandial glucose levels in response to a standard meal or oral glucose tolerance test, as well as mean 24-h glucose and glycemic excursions assessed using continuous glucose monitoring. In addition, fasting insulin decreases and improvement of insulin sensitivity were demonstrated. These changes were often accompanied by the decrease of blood triglyceride and cholesterol levels. However, a number of studies found that TRE had either adverse or no effects on glycemic and lipid traits, which might be explained by the different study designs (i.e., fasting/eating duration, daytime of eating, changes of calorie intake, duration of intervention) and study subject cohorts (metabolic status, age, gender, chronotype, etc.). To summarize, TRE represents an attractive and easy-to-adapt dietary strategy for the prevention and therapy of glucose and lipid metabolic disturbances. However, carefully controlled future TRE studies are needed to confirm these effects to understand the underlying mechanisms and assess the applicability of personalized interventions.

## Introduction

A growing body of evidence suggests that our circadian clock tightly interacts with metabolic functions (1) and that meal timing is an important factor for metabolic regulation (2–4). Chrononutrition, a novel discipline investigating the relation between circadian rhythms, nutrition, and metabolism, has developed rapidly in recent years (5). Chrononutrition clearly demonstrated that *when* we eat is as critical as *what* and *how much* for the chronic disease progression (3). The eating behavior in our modern society is often characterized by prolonged and erratic daily eating patterns which, together with Western-type diet, sedentary lifestyle, and chronic sleep deprivation, might contribute to an increased risk of obesity, diabetes, and cardiovascular diseases (6). In contrast, time-restricted eating (TRE), a dietary approach limiting the daily eating window, has attracted an increasing attention as an easy-to-use tool supporting weight loss, improving cardiometabolic health, and overall wellbeing (7–9). However, clinical trials on TRE demonstrated controversial effects especially in regard to glycemic and lipid traits.

This review focuses on the impact of TRE on glucose and lipid metabolism based on the clinical trials published until June 2021. We will also discuss different aspects of the TRE study design and different characteristics of study subjects as possible reasons for result bias. Further, we will shortly address molecular and physiological mechanisms of TRE effects. Finally, we will debate research gaps, which have to be filled by future studies and discuss the potential of TRE for the prevention and therapy of metabolic diseases.

## Tight Interaction of Circadian Clock and Metabolism

The endogenous circadian clock has been widely accepted to play an important role in the adaptation of the physiology and behavior of living organisms to the day-night changes, including in humans. In particular, circadian rhythms with a period of approximately 24 h regulate the metabolism of humans by synchronization of metabolic pathways, detaching non-compatible physiological and biochemical mechanisms, as well as improving the energy expenditure (1). In humans, circadian dysruptions resulting from shift work or chronic jet lag are related to obesity, metabolic syndrome, and cardiovascular diseases, which are consequences of an unbalanced metabolic homeostasis (10–12). Similar results were observed in animals with genetic knockout of key clock genes (13, 14). This leads to the assumption that metabolic health is preserved by proper functioning of circadian clocks. In turn, obesity and metabolic diseases, e.g., type 2 diabetes, alter or blunt circadian rhythms (15–17), confirming a tight reciprocal interaction of circadian clock and metabolism.

The circadian clock, in mammals, includes a master and a peripheral clock (1). The master clock is located at the hypothalamus in the suprachiasmatic nucleus (SCN). Peripheral oscillators orchestrated by the master clock are present in nearly every tissue, including metabolically active organs, such as the liver, adipose tissue, pancreas, and skeletal muscle. The molecular clock mechanism existing in almost every cell consists of interlocked transcriptional-translational feedback loops (2), including transcription factors *aryl hydrocarbon receptor nuclear translocator, like* (ARNTL, also known as BMAL1), *clock circadian regulator* (CLOCK), *period (*PER1, PER2, PER3*)*, *cryptochrome (*CRY1, CRY2*), retinoic acid-related orphan receptors (RORs)*, and *nuclear receptor subfamily 1 group D (NR1D1/2*, also known as *Rev-Erb*α/ß*)*. One cycle of this molecular machinery takes approximately 24 h and controls the expression of so-called clock-controlled genes (CCG), which include key metabolic transcription factors and enzymes generating circadian oscillations of metabolic functions (1, 2, 18). Indeed, 10% to 30% of the tissue transcriptome and 15% of circulating metabolome, as well as a number of circulating metabolic hormones—adipokines and cytokines involved in the regulation of carbohydrate, cholesterol, lipid, and energy metabolism—demonstrate circadian rhythms (19–24).

Notably, circadian clock itself undergoes metabolic and nutritional regulation. The reason is that food and feeding regimens are external cues (Zeitgeber), which can adjust (entrain) circadian rhythms in peripheral tissues. In mice fed with high-fat diet (HFD) and in mouse models of obesity, altered rhythms of core clock and a reorganisation of whole circadian transcriptome were observed (25–29). In humans, altered clock gene expression in human adipose tissue was found in obesity and metabolic syndrome (16, 17), and a blunted rhythm of clock gene expression in blood leucocytes was observed in type 2 diabetes (15). Our group showed in human intervention trials that both calorie intake and food composition affect circadian rhythms of clock and metabolic genes in blood monocytes and of circulating metabolic biomarkers (30, 31).

## Role of Meal Timing in Metabolic Regulation

Because of the tight interaction between the circadian clock and metabolism, timing of eating is an important parameter for modulating body weight and metabolic state. First evidence was obtained in mouse studies showing that animal feeding in the light (i.e., inactive) phase leads to a desynchronization between peripheral tissues and the central clock and induces weight gain and metabolic disturbances (32–34).

Studies involving humans provide similar results. Shift work or chronic jetlag and, as a result, consuming meals at the “wrong” or unusual time increase the risk of developing type 2 diabetes, cardiovascular diseases, and obesity (3, 12). This could be, at least in part, due to a lifestyle-induced discrepancy between sleep/wake, as well as fasting/feeding phase and internal circadian cycles, which may result in disruption of the fatty acid metabolism, glucose intolerance, and dysregulation of the body clock transcriptome as confirmed by experimental human studies on circadian misalignment (35–37). To note, other confounding factors, like sleep deprivation, decreased physical activity, or an unhealthy diet, may also negatively influence metabolism in case of shift work.

Notably, experimental human studies identified that parameters like glucose tolerance, insulin sensitivity, beta-cell responsiveness, and postprandial thermogenesis show better profiles in the morning than in the evening or afternoon (35, 38, 39). We and others demonstrated that meal consumption in the morning results in lower postprandial glucose concentrations and altered secretions of insulin, C-peptide, and of the incretins glucagon-like peptide 1 (GLP1) and gastric inhibitory polypeptide (GIP) compared to the consumption of the same meal in the afternoon (39–41). In healthy adults, late dinner (10 pm) causes shift in the postprandial period, overlapping with the sleep phase (42). Independent of this shift, higher glucose, a triglyceride peak delay, and lower free fatty acids and dietary fatty acid oxidation in the postprandial period were observed. Interestingly, late dinner did not affect sleep architecture, but increased plasma cortisol. Disturbances in circadian rhythms (alterations in the daily patterns of body-temperature and cortisol) were similarly observed in school-aged children consuming late dinner (43). Notably, the delay of meal timing or even change of the timely distribution of the calorie intake within the day without changing of sleeping times can shift expression rhythms of key clock genes in adipose tissue and blood cells (37, 44), possibly *via* postprandial hormone and metabolite changes.

In agreement with this, several human studies show that the timing of meals influences the outcome of weight loss therapy. Individuals consuming their lunch in the late hours lost less weight than the early eaters, although both groups consumed a hypocaloric diet (45). High caloric intake during breakfast has a positive effect on hunger scores, weight, as well as glucose, insulin, and ghrelin concentrations in comparison to the same intake during dinner (46). Late and delayed eating is associated with weight gain, dysfunction in energy expenditure, and abnormalities in the circadian rhythms of appetite, stress, and sleep hormones in most reports (47), although some epidemiological studies do not confirm these effects (48, 49). Notably, the night-eating syndrome clearly correlates with obesity (42). Furthermore, several experimental human studies show that eating in the evening worsens metabolic parameters in comparison with the improvement in daytime eaters (50–54).

Interestingly, novel studies suggest that certain time windows are more suitable for the consumption of certain kinds of food to maintain metabolic health. We recently showed that a consumption of high carb meals in the evening (in combination with high-fat meals in the morning) induces higher blood glucose levels and worsen glycemic control in subjects with an impaired glucose metabolism compared with a reverse pattern of meal composition (39). In agreement with this, epidemiological human studies report the positive effect of morning carbohydrate intake on the prevention of metabolic disorders (55, 56). Moreover, timing of carbohydrates and fat intake also affected circulating adipokine concentrations (57) and diurnal variation of the plasma lipidome (20).

Taken together, most published studies suggest that early eating is in alignment with our metabolic clock and therefore might be beneficial for metabolic health.

## Time-Restricted Eating: Idea, Definition, and Overview of Effects

In our modern society, prolonged and erratic daily eating patterns often take place. In American and Indian adults, eating periods of 15 h or longer every day were observed in more than half of the individuals. In addition, more than a third of the daily caloric intake occurred in the evening (6, 58). This eating period is often shifted to a later time on weekends indicating a “social jet lag” (59). Notably, reduction of the eating window in overweight individuals to 10 to 12 h resulted in sustained weight loss and improved subjective sleep quality after 16 weeks and 1 year of intervention (6). These data suggest that a shortening of the eating time and the accompanying elongation of the fasting time (≥ 12 h) might have beneficial effects on metabolic parameters in humans (60).

In the last years, time-restricted eating (TRE) has attracted increasing attention in public media as a strategy to lose weight and improve overall health. First data on TRE were collected in rodents where such diet is defined as “time-restricted feeding” (TRF). In mice, TRF increases the amplitude of circadian clock rhythms and is protective against HFD-induced obesity, glucose intolerance, leptin resistance, hepatic steatosis, and tissue inflammation compared with *ad libitum* HFD feeding (26, 61).

**Figure 1:**
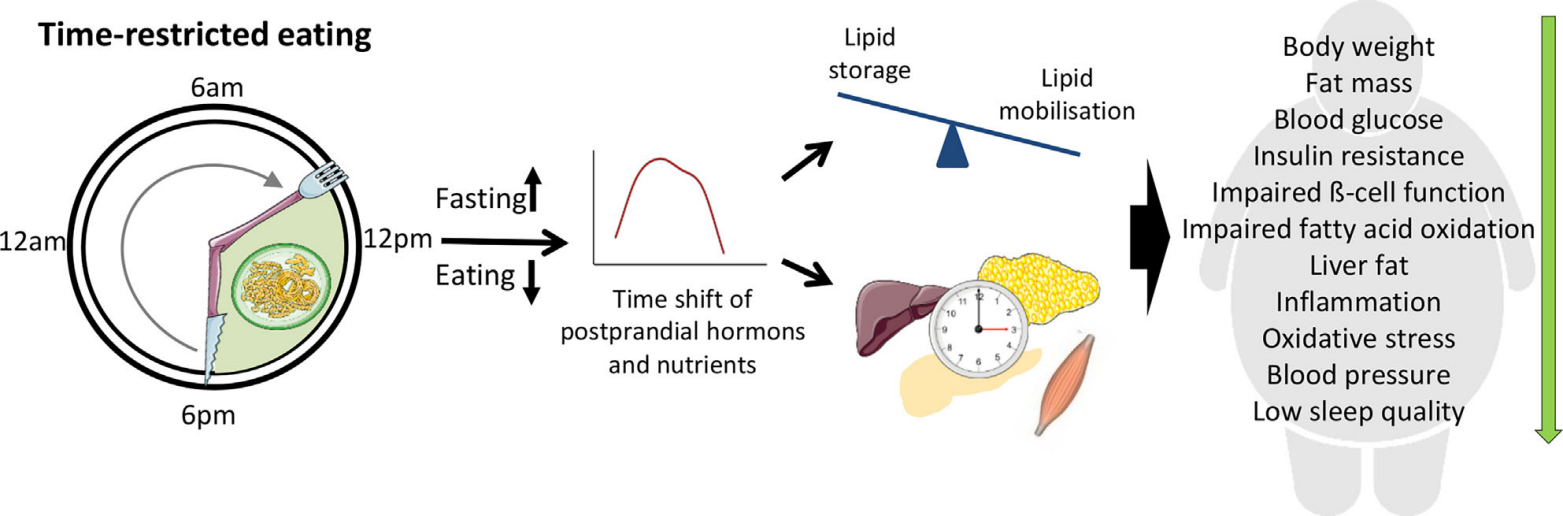
Beneficial effects of time-restricted eating (TRE) for individuals with metabolic disturbances. During TRE, elongation of the fasting period leads to the depletion of liver glycogen stores and a metabolic switch from lipid/cholesterol synthesis and fat storage to mobilization of fat through fatty acid oxidation and fatty acid-derived ketones. Modification of fasting–eating cycle can also directly influence peripheral clock which in turn contribute to the metabolic changes. The clock entrainment in peripheral tissues can be induced by the time shift of postprandial changes of metabolic hormones and nutrients acting *via* a number of molecular pathways as well as by the alterations of AMP/ATP ratio and cellular NAD+ availability.

In humans, the increased research interest to TRE initiated a number of intervention trials evaluating the effects of several TRE regimens with daily eating periods between 4 and 11 h on healthy individuals or participants with metabolic abnormalities (summarized in the **Table 1**). Most of them were short-term trials (4 days to 12 weeks) conducted in a relatively small number of subjects (8 to 80 participants) except for the study by Cai et al. with 174 subjects (63). In these studies, TRE not only appears to be a well-tolerated treatment strategy for overweight and obese patients but also generates beneficial metabolic effects (7, 59) (**Figure 1**), as discussed below. Most TRE studies reported modest reduction of body weight (6, 63–66, 68, 71–75, 77, 78, 82, 83, 85, 86), overall and visceral fat (62–65, 72, 74, 77, 78, 85, 86), and waist circumference (72, 73, 83, 86), which could be partly explained by the self-reported reduction of energy intake observed in many trials (6, 65, 66, 71, 82, 86, 87). Unexpectedly, TRE decreased feeling of hunger and desire to eat (76, 79, 80, 84) and the level of hunger hormone ghrelin (69, 70), although in one study fasting ghrelin level was increased (71) (**Table 1**). The data on satiety hormones, PYY and leptin, are also inconsistent (69, 70, 84), whereas adiponectin was increased in two studies (77, 78). TRE also reduced inflammatory markers (74, 78), blood pressure (67, 77, 84, 86) and oxidative stress markers (65, 84). In several studies, subjects reported an improvement of sleep quality, quality of life, and felt more energetic at the end of the intervention (6, 88). However, one study found no TRE effect on the gut microbiome (67), whereas another trial observed higher microbial diversity (87). Interestingly, TRE also affected gene expression of markers of the circadian clock, aging, and autophagy (69, 87).

**Table 1:** Clinical trials on TRE with outcomes regarding glucose and lipid metabolism.

Reference	Cohort (Male/Female)	Study design TRE Regimen(Fasting: Feeding)	Study duration	Calorie intake/weight change	Glucose metabolism	Lipid metabolism	Other effects
Antoni et al. 2018 (62)	n = 13 (1/12) healthy adults age: 29-57 years	non-randomized controlled trial parallel armTRE: daily feeding duration shortened by 3 h	12 weeks: 2 weeks baseline10 weeks intervention	− *Ad libitum* food access↓ daily energy intake ↔ distribution of macronutrients− ns body weight loss	↓ fasting glucose (primarily driven by an increase among controls)	− ns reduction in LDL and increase in HDL	↓ body fat mass
Cai et al., 2019 (63)	n = 174 (52/122) NAFLD patients age: 34,1 ± 6,6 years	RCT parallel arm TRE (16: 8) self-selected feeding window	12 weeks intervention	− *Ad libitum* food access− provided with a meal per day↔ energy intake↓ body weight	↔ fasting glucose and insulin	↓ serum TG↔LDL, HDL, and total cholesterol	↓ fat mass
Chow et al. 2020 (64)	n = 20 (3/17) overweight adults with a prolonged eating window >15 h/day age: 45,5 ± 12,1 years	RCT parallel arm TRE (13-16: 8-11) self-selected hour window	~16 weeks: ~ 4 weeks preintervention12 weeks intervention	− *ad libitum* food intake− no measure of caloric intake (or not published) ↓ body weight	*vs. preintervention*↓ fasting glucose↑ time blood glucose levels within target (70-180 mg/dL) (CGM measure)	*vs. preintervention*↓ TG↔ LDL, HDL	↔ physical activity↓ number of eating occasions*(a) vs. non-TRE group*↓ visceral fat↓ lean mass*(b) vs. preintervention*↓ fat mass ↓ lean mass↓ visceral fat
Cienfuegos et al., 2020 (65)	n = 49 (5/44) obese adults age: 47 ± 2 years	RCT parallel arm (a) lTRE (18: 6) 1 pm–7 pm.(b) lTRE (20: 4) 3 pm to 7 pm	10 weeks: 2 weeks baseline8 weeks intervention	− *ad libitum* food intake↓ energy intake ~550 kcal/day↓ body weight	↔ fasting glucose↓ fasting insulin↓ insulin resistance(partly driven by a worsening in control group)	↔ TG, HDL, and LDL	↓ fat mass↓ lean mass with (18: 6) vs. control and vs. (20: 4) ↓ 8-isoprostane
Gabel et al., 2018 (66) Gabel et al., 2020 (67)	n = 23 (3/20) obese adults age: 50 ± 2 years	historically controlled study TRE (16: 8) 10 am–6 pm	14 weeks: 2 weeks baseline12 weeks intervention	− *ad libitum* food intake↓ caloric intake~ 350 kcal/day↔ macronutrients↓ body weight	↔ glucose, insulin, HOMAR-IR	↔ TG, HDL, and LDL	↓ systolic blood pressure ↔ physical activity↔ gut microbiome
Hutchison et al., 2019 (68)	n = 15 (15/0) prediabetic men age: 55 ± 3 years	RCT crossover design (a) eTRE (15: 9) 8 am to 5 pm (b) lTRE (15: 9) 12 pm–9 pm	5 weeks: 1 week baseline 1 week each intervention 2 weeks washout	− *ad libitum* food intake− no measure of caloric intake (or not published) ↓ body weight	↓ glucose iAUC↔ fasting glucose and insulin↓ mean fasting glucose in eTRE (CGM data)	↓ fasting TG↔ NEFA	↔ physical activity ↔ gastrointestinal hormones ↔ perceived hunger, fullness, desire to eat
Jamshed et al., 2019 (69) Ravussin et al., 2019 (70)	n = 11 (7/4) overweight adults age: 32 ± 7 years	RCT crossover design eTRE (18: 6) 8 am–2 pm	~5-6 weeks: 4 days each intervention 3,5-5 weeks washout	− isocaloric controlled feeding approach− days 1-2: participants followed eating schedule on their own− days 3-4: standardized meals eaten under supervision↔ weight between arms before respiratory chamber↓ body weight while respiratory chamber day in eTRE vs. controls	↓ mean 24-h glucose ↓ glycemic excursions (M age) ↓ morning fasting glucose, insulin, and HOMA-IR ↑ evening insulin and HOMA-IR↔ evening glucose	↑ morning total cholesterol, HDL, LDL↔ morning TG, free fatty acids↔ evening lipid levels	↔ 24 h energy expenditure ↓ mean ghrelin↓ morning levels of ghrelin, leptin, GLP-1↑ PYY in evening↑ metabolic flexibility ↑ ketones in morning↓ evening cortisol
Jones et al. 2020 (71)	n = 16 (16/0) healthy men age: 23 ± years	Non-randomized trial Two groups recruited & tested temporally apart(a) eTRE (16: 8) 8 am–4 pm(b) energy-matched control/caloric restriction intervention (CON : CR)	3 weeks: 1 week baseline 2 weeks intervention	− *ad libitum* food intake in eTRE group− prescribed dietary plans and all dietary intakes provided in CON : CR group to match eTRE↓ caloric intake~ 400 kcal/day↓ body weight (matched in Con : CR)	↑ whole-body insulin sensitivity↑ glucose uptake of skeletal muscle↔ mean 24-h glucose↔ fasting insulin	↔ serum TG	↔ physical activity↑ fasting ghrelin
Karras et al., 2020 (72)	n_total_ = 60 (17/43) orthodox fasting n=37 (11/26) TRE n=23 (6/17) overweight, metabolically healthy adults age: 48.3 ± 8.9 years	non-randomized, parallel arm trial(a) orthodox fasting (b) eTRE (16: 8) 8 am–4 pm	12 weeks: 7 weeks intervention follow up 5 weeks after intervention	(a) hypocaloric dietary plans based on orthodox fasting: no animal products, except for 2 days fish (b) hypocaloric dietary plans with two meals (8 am and 1 pm) and two snacks (11 am and 3: 30 pm) ↔ caloric intake↓ body weight (both groups vs. pre-intervention)	↔ fasting glucose, fasting insulin↔ insulin resistance (HOMA-IR) and ß-cell function	↔ total cholesterol, LDL↑ TG↓ HDL	*vs. orthodox fasting: *↓ waist circumference*vs. pre-intervention: *↓ waist circumference↓ body fat↔ C-reactive protein
Kesztyüs et al., 2019 (73)	n = 40 (9/31) abdominally obese adults age: 49,1 ± 12,4 years	single arm trial TRE (15-16: 8-9) self-selected hour window	12 weeks intervention	− *ad libitum* food intake− no measure of caloric intake↓ body weight	↓ HbA1c	↔ total cholesterol, HDL, LDL, TG	↓ waist circumference
Li et al., 2021 (74)	n = 15 (0/15) women with anovulation and PCOS age: between 18 and 31 years	single arm triale TRE (16: 8) 8 am–4 pm	6 weeks: 1 week baseline 5 weeks intervention	− isocaloric approach (fluctuations for no more than 10% from baseline caloric intake) ↓ body weight	↔ fasting glucose, glucose AUC↓ fasting insulin, insulin AUC↓HOMA-IR, AUC_Insulin_/AUC_Glucose_	↔ total cholesterol, LDL, TG	↓ body fat↓ visceral fat↓ C-reactive protein and alanine aminotransferase↑ IGF-1
Lowe et al. 2020 (75)	n_total_ = 116 (70/46) in-person tested: n = 50 (28/22) overweight, obese adults age: 46,5 ± 10,5 years	RCT parallel arml TRE (16: 8) 12 pm–8 pm	12 weeks intervention	− *ad libitum* food intake− no measure of caloric intake↓ body weight (*vs.* baseline ns from control group)	↔ fasting glucose, fasting insulin, HOMA-IR, HbA1c	↔ total cholesterol, HLD, LDL, TG	↓ appendicular lean mass
Martens et al., 2020 (76)	n = 22 (10/12) healthy, non-obese adults age: 67 ± 1 years	RCT crossover design lTRE (16: 8) consistent self-selected hour window starting between 10 and 11 am	7 weeks: 1 week baseline 6 weeks each intervention	− *ad libitum* food intake↔ total energy intake↔ diet composition (healthy eating index) ↔ body weight	↔ fasting glucose↓ glucose AUC (during OGTT) ↔ plasma insulin	↑ total cholesterol and LDL	↔ total body composition ↔ bone density↓ sensation of hunger
McAllister et al., 2019 (77)	n = 22 (22/0) physically active men age: 22 ± 2,5 years	randomized two parallel arm trial (a) *ad libitum* TRE (16: 8) (b) isocaloric TRE (16: 8) self-selected hour window	4 weeks intervention	(a) *ad libitum* food intake (b) isocaloric food intake = advised to eat <± 300 kcal from baseline measured caloric intakein both groups: − ns lower caloric intake↓ body weight	↔ blood glucose↔ plasma insulin	↑ HDL↔ mean TG	↓ body fat↓ blood pressure ↑ adiponectin
Moro et al., 2016 (78)	n = 34 (34/0) healthy resistance trained men age: 29,21 ± 3,8 years	RCT parallel arm (a) lTRE (16: 8) 1 pm–9 pm+ RT (b) control eating window: 8 am–9 pm + RT	8 weeks intervention	− caloric intake consumed in 3 meals per day at standardized times within 1 h↔ caloric intake and macronutrient distribution↓ body weight	*TRE pre vs. post (not control) : *↓ blood glucose ↓ insulin andHOMA-IR	↓ TG↔ total cholesterol, LDL, HDL	↓ fat mass↔ lean mass ↓ testosterone↓ IGF-1 ↑ adiponectin↓TNF-α, IL-1β ↓ respiratory ratio
Parr et al. 2020 (79)	n = 19 (9/10) adults with T2D and eating window >12 h/day age: 50 ± 9 years	single arm trial TRE (15: 9) 10 am–7 pm	6 weeks: 2 weeks baseline4 weeks intervention	− *ad libitum* food intake↔ caloric intake and macronutrient distribution↔ body weight	↔ fasting glucose, insulin ↔ glucose and insulin AUC ↔ HbA1c	↔ total cholesterol, HDL, LDL, TG	↑ adherence to TRE reduced energy intake ↔ psychological well-being
Parr et al., 2020 (80) Lundell et al. 2020 (81)	n = 11 (11/0) sedentary men with overweight/obesity age: 38 ± 5 years	RCT crossover design (a) TRE (16: 8) 10 am–6 pm (b) extended feeding of 15 h/day7 am–10 pm	3 weeks: 5 days each intervention 10 days washout	− isocaloric feeding, meals provided and consumed at standardized times within ± 30 min− no measure of weight loss	↓ nocturnal glucose AUC ↔ waking glucose AUC ↔ peak glucose↔ 24-h and peak glucose (CGM) ↔ peak insulin− ns tendency to ↓ AUC _total_ insulin	↔ TG AUC _total_↑ peak TG↑ AUC _total_ and peak NEFA	↓ C-Peptide AUC _total_↔ physical activity↓ evening hunger ↔ muscle core clock gene expression− alters rhythmicity of serum and muscle metabolites and amino acid transport
Peeke et al., 2021 (82)	n = 60 (7/53) obese adults age: 44 ± 11 years	RCT parallel arm, virtual trial (a) control: (12: 12) (b) TRE: (14: 10) with fasting snack after 12 h on 5 days/weekin both groups fasting period began after dinner between 5 pm and 8 pm	8 weeks intervention	− Hypocaloric dietary regimes based on Jenny Craig^®^ Rapid Results program− 3 meals and 1 snack provided per day↓ body weight	↓ fasting plasma glucose	n/a	Fasting snack decreased hunger
Phillips et al., 2021 (83)	n = 45control n=20adults with eating windows ≥14 h/day and at least one metabolic syndrome component age: 43.4 ± 13.3 years	RCT parallel arm TRE (12: 12) self-selected hour window	7 months: 4 weeks baseline6 months intervention	− *ad libitum* food intake− no quantitative measure of caloric intake↓ body weight	↔ fasting glucose, HbA1c	↔ HDL cholesterol, TG	↓ waist circumference
Sutton et al. 2018 (84)	n = 8 (8/0) overweight men with prediabetes age: 56 ± 9 years	RCT crossover design eTRE (18: 6) dinner before 3 pm	17 weeks: 5 weeks each intervention7 weeks washout	− isocaloric controlled feeding approach with standardized meals eaten under supervision− ns weight loss	↓ insulin (fasting, mean and peak) ↑ insulin sensitivity↑ß-cell responsiveness↓ insulin resistance	↑ fasting TG↔ HDL and LDL cholesterol	↓blood pressure↓desire to eat in the evening↓8-isoprostane↓fasting PYY
Tinsley et al. 2019 (85)	ITT: n = 40 (0/40) PP: n = 24 (0/24) healthy resistance trained females age: 22,1 ± 2,6 years	RCT parallel arm (a) lTRE (16: 8) 12 pm–8 pm + placebo +RT(b) lTRE (16: 8) 12 pm–8 pm + 3 mg/day HMB + RT(c) ND + placebo + RT	8 weeks intervention	− *ad libitum* food intake+ provided supplemental whey protein↔ caloric intake (increase in all groups due to provided protein supplements) ↓ body weight	↔ fasting glucose, insulin	↔ total cholesterol, HLD, LDL, TG	↔ muscular improvements↓ fat mass in PP analysis in lTRE groups
Wilkinson et al., 2020 (86)	n = 19 (13/6) adults with metabolic syndrome and eating window ≥14 h/day age: 59 ± 11 years	single arm trial TRE (14: 10) consistent self-selected hour window	14 weeks: 2 weeks baseline12 weeks intervention	− *ad libitum* food intake↓ caloric intake↓ body weight	ns trend towards↓ fasting glucose, fasting insulin, and HbA1c↓ HbA1c in participants with elevated fasting glucose and HbA1c at baseline	↓ total cholesterol, LDL, and non-HDL↔ TGns tendency to lower HDL	↓ body fat↓ visceral fat↓ waist circumference↓ blood pressure ↑ restfulness↔ physical activity
Zeb et al., 2020 (87)	n = 80 (80/0) control n=24young aged healthy men age: n/a	RCT parallel arml TRE (16: 8) 7: 30 pm–3: 30 am	25 days intervention	− *ad libitum* food intake↓ caloric intake↔ body weight	n/a	↓ total cholesterol and TG↔ LDL ↑ HDL (in both: pre- *vs.* post-intervention and *vs.* control)	↑ liver function↑ gut microbial diversity↑ circadian gene expression (*Bmal1, Clock, Sirt1*)

Studies investigated effects of a single meal consumed at different times of the day are not included in the table. ↑, increase; ↓, decrease; ↔, no significant change. AUC_total_, total 24-h area under the curve; CGM, continuous glucose monitoring; CON, CR, control/caloric restriction intervention; eTRE, early time-restricted eating; EXF, extended feeding; HbA1c, glycated hemoglobin A1c; HDL, high density lipoprotein; HMB, β-hydroxy β-methylbutyrate; HOMA-IR, homeostatic model assessment of insulin resistance; iAUC, incremental area under the curve; ITT, intention to treat; LDL, low density lipoprotein; lTRE, late time-restricted eating; MAGE, mean amplitude of glycemic excursions; ND, normal diet; NEFA, nonesterified fatty acids; ns, non-significant modification (p > 0,05); OGTT, oral glucose tolerance test; PCOS, polycystic ovary syndrome; PP, per protocol; PYY, peptide YY; RCT, randomized controlled trial; RT, resistance training; T2D, type 2 diabetes; TG, triglycerides; TRE, time-restricted eating; n/a, not available.

Furthermore, many TRE trials demonstrated improvement of glucose metabolism and blood lipids, whereas other studies found no or adverse effects of TRE on glycemic and lipid traits which will be discussed in detail in the next two chapters.

## Effects of TRE on Glucose Metabolism

Eight studies revealed positive changes in glucose levels because of TRE (62, 64, 68, 69, 76, 78, 80, 82), although this was not universally observed (63, 65, 66, 72, 74, 75, 77, 79, 83, 85). Nonetheless, TRE interventions significantly lowered fasting glucose levels (62, 64, 69, 78, 82), postprandial glucose levels in response to a standard meal or oral glucose tolerance test (OGTT) (68, 76), and night-time glucose (80). Few TRE trials applied continuous glucose monitoring (CGM) to monitor the glucose concentration over the 24 h per day as opposed to only certain time points. The beneficial effects appear even in CGM data, e.g., decreased mean 24-h glucose, glycemic excursions (69), and mean fasting glucose (68). HbA1c, as an important indicator of long-term glycemic control, decreased in two trials after TRE intervention in overweight and obese participants (73, 86), whereas no changes were detected in three other studies (75, 79, 83). Moreover, TRE could be shown to significantly lower fasting insulin levels, as well as insulin resistance (65, 69, 74, 78, 84) and increase insulin sensitivity (71, 84). Again, these results are not consistent in all conducted studies as other TRE interventions had no notable effect on insulin levels or insulin resistance (63, 66, 68, 72, 75–77, 79, 80, 85).

Improvement of glucose metabolism with TRE could be, at least in part, due to caloric restriction and associated weight loss (7, 8) (**Table 1**). Nevertheless, four TRE trials revealed beneficial effects on the glucose metabolism, regardless of caloric restriction or weight loss (62, 69, 76, 84). These findings suggest that the TRE dietary habit could produce positive metabolic effects, independent of energy balance.

## Effects of TRE on Lipid Metabolism

Observed effects of TRE on plasma lipid levels are highly variable. Therefore, the triglyceride (TG) levels decreased significant in five TRE-studies (63, 64, 68, 78, 87), whereas no significant changes in TG levels occurred in 12 other studies (65, 66, 69, 71, 73–75, 77, 79, 83, 85, 86), and in two isocaloric trials, the fasting TG concentration actually increased (72, 84). Concerning cholesterol levels (total, HDL and LDL), the current state of improvements through TRE is inconsistent. In most TRE interventions, no significant changes of cholesterol levels emerged (**Table 1**). Nevertheless, in some cases, the shortened time of caloric intake improved participants cholesterol levels, by either decreasing total cholesterol and LDL (86, 87) or notably increasing HDL blood concentrations (77, 87). Contrarily, in one study, the TRE protocol leads to an increase of total and LDL cholesterol (76). Similar to glucose metabolism, TRE can affect lipid metabolism regardless of weight loss/caloric restriction (76, 84, 87).

The present work indicates that the effects of TRE on glycemic control are apparently less variable than effects of TRE on plasma lipid profiles. One possible explanation of this phenomenon might be the known diurnal rhythm of glucose tolerance, which decreases from morning to evening and night, whereas it is established that lipid circadian rhythms show substantial inter-individual variability (89).

## Current Research Gaps and Open Questions

Taken together, TRE represents a promising and simple dietary approach for the prevention and therapy of disturbances in glucose and lipid metabolism (e.g., obesity, type 2 diabetes, metabolic syndrome, etc.) in the general population (because it does not require extensive nutritional knowledge) and medical practice. Published TRE studies included several different subject groups varying from lean and healthy adults (62, 71, 76–78, 87), overweight, and obese subjects (64–66, 69, 70, 72, 73, 75, 80, 82) up to adult individuals with metabolic disturbances such as prediabetes (68, 84), type 2 diabetes (79), metabolic syndrome (83, 86), or NAFLD (63). These discrepancies between study participants should be noted as it is more likely to observe metabolic benefits in cohorts with obesity or pre-existing disturbances of lipid and/or glucose metabolism compared to healthy lean adults. However, some studies reported metabolic improvements also in healthy subjects, e.g., improved glucose levels, insulin sensitivity (71, 78), or blood lipid levels (77, 78, 87), indicating that TRE could be beneficial even for healthy individuals. However, there are some groups of individuals for which the TRE may not be appropriate for, e.g., children and teenagers who are actively growing, subjects taking drugs or insulin injections to lower blood sugar, especially people with type 1 diabetes, subjects with acute illness, eating disorders, pregnancy or are breastfeeding, as well as individuals with severe kidney and liver diseases and cancer. TRE might be harmful to these subjects, consequently they should consider TRE only in consultation with and supervision by a physician.

Further, there is a range of research gaps and open questions in this field of TRE research, which need further investigation. The main question raised is the reason for the inconsistencies in metabolic outcomes between TRE studies. This might be explained by the different study designs (e.g., fasting/eating duration, daytime of eating, changes of calorie intake, duration of intervention) and study subject cohorts (metabolic status, age, gender, chronotype, etc.) as already mentioned above. The next important limitation is that the wide spectrum of TRE regimens with daily eating periods between 4 and 11 h and food consumption early (eTRF) or late in the day (lTRE) makes the dietary effects scarcely comparable. In particular, optimal duration of the eating window is unknown, suggesting that a direct comparison of varying eating windows (e.g., 6 h *vs* 9 h *vs*. 12 h) has to be performed in the future studies upon the careful controlling of caloric intake as discussed below.

Early morning is likely to be an optimal TRE time to induce maximal metabolic benefits. In most eTRE studies, restricting food intake to the morning resulted in an improvement of insulin sensitivity, beta-cell responsiveness, blood pressure, inflammation, and oxidative stress (69–71, 84). In contrast, on lTRE, restricting food intake to the late afternoon or evening (after 4 pm) did not change or even worsen blood glucose, beta-cell responsiveness, and lipid levels (65, 75, 76, 78, 85). Notably, until now, only one study directly compared eTRE and lTRE in a cross-over design (68) where postprandial glucose and fasting triglycerides decreased after consuming both diets, whereas mean fasting glucose assessed by CGM improved only with eTRE. Trials in which eating window was restricted to the middle of the day or self-defined window but was not precisely matched, resulted in a reduced body weight or fat mass, with contradictory results concerning fasting glucose, insulin, and lipids (6, 66, 80, 81, 86). This, in combination with generally small sample sizes (and correspondingly low power) and lack of long-term interventions makes published results hard to interpret and to formulate dietary recommendations.

In most trials that investigated TRE under free-living conditions, participants were adherent to the prescribed eating windows on more than 80% of days throughout the intervention period (63–66, 71, 73, 75, 76, 86). Only Parr et al. (79) reported adherence rates to a 9-h TRE intervention to be minimally lower, with 72 ± 24%. In summary, the adherence to TRE over short periods is high, suggesting that TRE is a feasible and easy-to-adapt dietary strategy. However, the long-term practicality of a dietary approach is crucial for beneficial health outcomes (90), and therefore, future trials should examine the adherence to TRE in real life settings over longer periods.

Notably, implementing of eTRE may be challenging for general population because the meals in the evening, consumed after work, are an important family and social event. Late TRE leading to skip breakfast would be better compatible with social life, but is less effective or can even induce adverse metabolic effects as mentioned above. Moreover, shifting of the meal time to a later time of day can induce clock phase delay in peripheral tissues (50); however, metabolic consequences still needs to be further investigated. Thus, timing of meals is considerably associated with quality of life and TRE adherence. Until now, only several trials analyzed individual’s life quality on TRE and reported its improvement when using self-defined eating windows (6, 88). Future studies comparing effects of various TRE windows on the life quality are needed. The next question is whether individual’s chronotype has to be considered when prescribing optimal eating times. Chronotype is a behavioral manifestation of an individual’s internal clock; and *late chronotype* (“owls”) and *early chronotype* (“larks”) are the two extremes which strongly differ in peak times of metabolic function, body temperature, cognitive faculties, and sleeping (59) as well as eating habits (91, 92). Whether late chronotypes can profit in the same matter from the eTRE as early chronotypes or the lTRE is more suitable for such individuals also needs future investigation.

The further important question is whether beneficial metabolic effects of TRE and even the weight loss are resulting from the reduction of energy intake alone or also from the shortening of the eating window (and corresponding prolongation of fasting). Most published trials reported a reduction of energy intake because individuals are often not able to consume all usual food quantity within the limited time window. Nevertheless, four carefully controlled or short-term (4–5 days) TRE trials revealed beneficial effects on the glucose metabolism without caloric restriction or weight loss (62, 69, 76, 84), suggesting that timing factor alone can improve metabolic state. Further, most of the published human TRE studies did not carefully monitor dietary macronutrient content, which could lead to false data interpretation, e.g., if subjects have to skip high-fat or sweet snacks or alcohol drinks often consumed in the evening.

This opens a next question concerning physiological and molecular mechanisms underlying metabolic TRE effects. One possible explanation of this effect might be the elongation of the fasting period (typically beyond 12 h), which leads to the depletion of liver glycogen stores and a metabolic switch from lipid/cholesterol synthesis and fat storage to mobilization of fat through fatty acid oxidation and fatty acid-derived ketones (60). Further, prolonged fasting might improve metabolism and reduce oxidative stress *via* autophagy activation, although relevant publications are sparse. In rodents, intermittent fasting regimen with 24-h fasting periods preserved beta-cell mass in obesity-induced diabetes *via* the autophagy–lysosome pathway (93) and a similar mechanism might work for shorter fasting duration. In humans, only one study investigated TRE effects on autophagy genes and found changes of *LC3A* and *ATG12* expression in whole blood (69). Some data also suggest that the modification of fasting–eating cycle is likely to influence peripheral clock itself which in turn contribute to the metabolic changes. The clock entrainment in peripheral tissues such as liver or adipose tissue can be induced by postprandial changes of metabolic hormones, i.e., insulin and oxyntomodulin (94, 95). Moreover, the postprandial increase of glucose, lipids, and amino acids may affect the circadian clock *via* key intracellular metabolic sensors such as SIRT1, mTOR, S6K, AMPK, PPARs, RORs, and Rev-Erbs (96). In particular, fasting increases AMP/ATP ratio and cellular availability of NAD+, regulating clock machinery *via* AMPK and SIRT1, respectively (8). Notably, the careful timing of the physical activity in the context of TRE could intensify its metabolic effects because several common mechanisms are activated by exercise and prolonged fasting (97). However, most of these mechanisms were described in rodents and require intensive investigation in human TRE studies. In particular, it is unknown whether eTRE is more beneficial than lTRE for the synchronization and improvement of clock rhythms in humans. Further analyses of novel biomarkers (e.g., adipokines, cytokines, oxidative stress markers, gene expression in muscle or adipose tissue) and using modern techniques and portable devices for the continuous monitoring of glucose, physical activity, sleep quality, and food intake (e.g. using smartphones) would provide new data on physiological and molecular mechanisms induced by TRE.

Taken together, further human trials are needed to investigate effects of TRE: (1) carefully monitoring macronutrient and calorie intake (possibly *via* conducting an isocaloric TRE); (2) directly comparing effects of eTRE and lTRE; (3) comparing varying eating window duration; (4) in long-term studies; (5) in a large number of study participants; (6) comparing TRE effects in subjects with different chronotypes; (7) including analyses of physiological and molecular mechanisms underlying the TRE-induced changes. In particular, calling for research that balances feasibility of TRE interventions (e.g., timing and duration of eating windows) with long-term adherence and metabolic benefits could be recommended.

Confirming a large scientific and practical interest to the TRE approach, there are more than 20 ongoing intervention trials applying TRE approaches to improve body weight and metabolic state of individuals as based on published study protocols and a search in ClinicalTrials.gov database. A few trials will be conducted in larger cohorts with more than 100 participants, however several trials are planned in smaller cohorts with well-defined participants, e.g., with specific medical conditions linked to obesity. Moreover, most interventions are still short term with intervention periods lasting 2 to 12 weeks, and only eight studies scheduled longer interventions (up to 1 year). Solely, four trials will be directly comparing TRE at different daytimes. Moreover, none of the ongoing trials aims to compare TRE in individuals with different chronotypes, although the subjects’ chronotype will be assessed in several studies. In consequence, even though many TRE trials are ongoing, it remains unclear if these trials will be sufficient to answer all the abovementioned research gaps and formulate dietary recommendations for the general public.

## Conclusions and Perspectives

TRE represents an attractive and easy-to-adapt dietary strategy for the prevention and therapy of glucose and lipid metabolic disturbances. It might be widely used to restore disturbed circadian rhythms and to improve metabolic health in obesity, insulin resistance, metabolic syndrome, and cardiovascular diseases. In the best way, TRE approach has to be used in combination with healthy dietary composition, an increased physical activity, and adequate sleep quality and duration to support optimal health. However, future carefully controlled TRE studies are needed to formulate dietary recommendations for the general population and medical practice.

## Author Contributions

OP-R generated the idea and performed the supervision of the manuscript preparation. All authors contributed to the article and approved the submitted version.

## Funding

The study was supported by a grant of the German Science Foundation (DFG RA 3340/3-1 OP-R), of the German Diabetic Association (Allgemeine Projektförderung der DDG 2020, OP-R; and Adam-Heller-Projektförderung der DDG/Abbott, 2021, OP-R), and by the Morgagni Prize of the European Association of Study of Diabetes 2020 (OP-R).

## Conflict of Interest

The authors declare that the research was conducted in the absence of any commercial or financial relationships that could be construed as a potential conflict of interest.

## Publisher’s Note

All claims expressed in this article are solely those of the authors and do not necessarily represent those of their affiliated organizations, or those of the publisher, the editors and the reviewers. Any product that may be evaluated in this article, or claim that may be made by its manufacturer, is not guaranteed or endorsed by the publisher.

## References

[1] Panda S. Circadian Physiology of Metabolism. Science (2016) 354:1008–15. 10.1126/science.aah4967 PMC7261592 27885007

[2] Asher G Sassone-Corsi P. Time for Food: The Intimate Interplay Between Nutrition, Metabolism, and the Circadian Clock. Cell (2015) 161:84–92. 10.1016/j.cell.2015.03.015 25815987

[3] Jiang P Turek FW. Timing of Meals: When Is as Critical as What and How Much. Am J Physiol Endocrinol Metab (2017) 312:E369–80. 10.1152/ajpendo.00295.2016 PMC6105931 28143856

[4] Kessler K Pivovarova-Ramich O. Meal Timing, Aging, and Metabolic Health. Int J Mol Sci (2019) 20:1911. 10.3390/ijms20081911 PMC6514931 31003407

[5] Johnston JD Ordovas JM Scheer FA Turek FW. Circadian Rhythms, Metabolism, and Chrononutrition in Rodents and Humans. Adv Nutr (2016) 7:399–406. 10.3945/an.115.010777 26980824 PMC4785478

[6] Gill S Panda S. A Smartphone App Reveals Erratic Diurnal Eating Patterns in Humans That Can Be Modulated for Health Benefits. Cell Metab (2015) 22:789–98. 10.1016/j.cmet.2015.09.005 PMC4635036 26411343

[7] Adafer R Messaadi W Meddahi M Patey A Haderbache A Bayen S. Food Timing, Circadian Rhythm and Chrononutrition: A Systematic Review of Time-Restricted Eating’s Effects on Human Health. Nutrients (2020) 12:3770. 10.3390/nu12123770 PMC7763532 33302500

[8] Regmi P Heilbronn LK. Time-Restricted Eating: Benefits, Mechanisms, and Challenges in Translation. iScience (2020) 23:101161. 10.1016/j.isci.2020.101161 32480126 PMC7262456

[9] Chaix A Manoogian ENC Melkani GC Panda S. Time-Restricted Eating to Prevent and Manage Chronic Metabolic Diseases. Annu Rev Nutr (2019) 39:291–315. 10.1146/annurev-nutr-082018-124320 31180809 PMC6703924

[10] Vetter C Devore EE Wegrzyn LR Massa J Speizer FE Kawachi I. Association Between Rotating Night Shift Work and Risk of Coronary Heart Disease Among Women. JAMA J Am Med Assoc (2016) 315:1726–34. 10.1001/jama.2016.4454 PMC5102147 27115377

[11] De Bacquer D Van Risseghem M Clays E Kittel F De Backer G Braeckman L. Rotating Shift Work and the Metabolic Syndrome: A Prospective Study. Int J Epidemiol (2009) 38:848–54. 10.1093/ije/dyn360 19129266

[12] Antunes LC Levandovski R Dantas G Caumo W Hidalgo MP. Obesity and Shift Work: Chronobiological Aspects. Nutr Res Rev (2010) 23:155–68. 10.1017/S0954422410000016 20122305

[13] Turek FW. Obesity and Metabolic Syndrome in Circadian Clock Mutant Mice. Science (2005) 308:1043–5. 10.1126/science.1108750 PMC3764501 15845877

[14] Paschos GK Ibrahim S Song WL Kunieda T Grant G Reyes TM. Obesity in Mice With Adipocyte-Specific Deletion of Clock Component Arntl. Nat Med (2012) 18:1768–77. 10.1038/nm.2979 PMC3782286 23142819

[15] Ando H Takamura T Matsuzawa-Nagata N Shima KR Eto T Misu H. Clock Gene Expression in Peripheral Leucocytes of Patients With Type 2 Diabetes. Diabetologia (2009) 52:329–35. 10.1007/s00125-008-1194-6 18974966

[16] Vieira E Ruano E Figueroa AL Aranda G Momblan D Carmona F. Altered Clock Gene Expression in Obese Visceral Adipose Tissue Is Associated With Metabolic Syndrome. PloS One (2014) 9:e111678. 10.1371/journal.pone.0111678 25365257 PMC4218799

[17] Gomez-Abellan P Hernandez-Morante JJ Lujan JA Madrid JA Garaulet M. Clock Genes Are Implicated in the Human Metabolic Syndrome. Int J Obes (Lond) (2008) 32:121–8. 10.1038/sj.ijo.0803689 17653067

[18] Brown SA. Circadian Metabolism: From Mechanisms to Metabolomics and Medicine. Trends Endocrinol Metabolism: TEM (2016) 27:415–26. 10.1016/j.tem.2016.03.015 27113082

[19] Keller M Mazuch J Abraham U Eom GD Herzog ED Volk HD. A Circadian Clock in Macrophages Controls Inflammatory Immune Responses. Proc Natl Acad Sci USA (2009) 106:21407–12. 10.1073/pnas.0906361106 PMC2795539 19955445

[20] Kessler K Gerl MJ Hornemann S Damm M Klose C Petzke KJ. Shotgun Lipidomics Discovered Diurnal Regulation of Lipid Metabolism Linked to Insulin Sensitivity in Nondiabetic Men. J Clin Endocrinol Metab (2020) 105:dgz176. 10.1210/clinem/dgz176 31680138

[21] Held NM Wefers J van Weeghel M Daemen S Hansen J Vaz FM. Skeletal Muscle in Healthy Humans Exhibits a Day-Night Rhythm in Lipid Metabolism. Mol Metab (2020) 37:100989. 10.1016/j.molmet.2020.100989 32272236 PMC7217992

[22] Loboda A Kraft WK Fine B Joseph J Nebozhyn M Zhang C. Diurnal Variation of the Human Adipose Transcriptome and the Link to Metabolic Disease. BMC Med Genomics (2009) 2:7. 10.1186/1755-8794-2-7 19203388 PMC2647943

[23] Dallmann R Viola AU Tarokh L Cajochen C Brown SA. The Human Circadian Metabolome. Proc Natl Acad Sci USA (2012) 109:2625–9. 10.1073/pnas.1114410109 PMC3289302 22308371

[24] Christou S Wehrens SMT Isherwood C Moller-Levet CS Wu H Revell VL. Circadian Regulation in Human White Adipose Tissue Revealed by Transcriptome and Metabolic Network Analysis. Sci Rep (2019) 9:2641. 10.1038/s41598-019-39668-3 30804433 PMC6389935

[25] Kohsaka A Laposky AD Ramsey KM Estrada C Joshu C Kobayashi Y. High-Fat Diet Disrupts Behavioral and Molecular Circadian Rhythms in Mice. Cell Metab (2007) 6:414–21. 10.1016/j.cmet.2007.09.006 17983587

[26] Hatori M Vollmers C Zarrinpar A DiTacchio L Bushong EA Gill S. Time-Restricted Feeding Without Reducing Caloric Intake Prevents Metabolic Diseases in Mice Fed a High-Fat Diet. Cell Metab (2012) 15:848–60. 10.1016/j.cmet.2012.04.019 PMC3491655 22608008

[27] Eckel-Mahan KL Patel VR de Mateo S Orozco-Solis R Ceglia NJ Sahar S. Reprogramming of the Circadian Clock by Nutritional Challenge. Cell (2013) 155:1464–78. 10.1016/j.cell.2013.11.034 PMC4573395 24360271

[28] Su W Xie Z Guo Z Duncan MJ Lutshumba J Gong MC. Altered Clock Gene Expression and Vascular Smooth Muscle Diurnal Contractile Variations in Type 2 Diabetic Db/Db Mice. Am J Physiol Heart Circulatory Physiol (2012) 302:H621–633. 10.1152/ajpheart.00825.2011 PMC3353796 22140039

[29] Ando H Kumazaki M Motosugi Y Ushijima K Maekawa T Ishikawa E. Impairment of Peripheral Circadian Clocks Precedes Metabolic Abnormalities in Ob/Ob Mice. Endocrinology (2011) 152:1347–54. 10.1210/en.2010-1068 21285316

[30] Pivovarova O Gogebakan O Sucher S Groth J Murahovschi V Kessler K. Regulation of the Clock Gene Expression in Human Adipose Tissue by Weight Loss. Int J Obes (Lond) (2016) 40:899–906. 10.1038/ijo.2016.34 26902807

[31] Pivovarova O Jurchott K Rudovich N Hornemann S Ye L Mockel S. Changes of Dietary Fat and Carbohydrate Content Alter Central and Peripheral Clock in Humans. J Clin Endocrinol Metab (2015) 100:2291–302. 10.1210/jc.2014-3868 25822100

[32] Damiola F. Restricted Feeding Uncouples Circadian Oscillators in Peripheral Tissues From the Central Pacemaker in the Suprachiasmatic Nucleus. Genes Dev (2000) 14:2950–61. 10.1101/gad.183500 PMC317100 11114885

[33] Arble DM Bass J Laposky AD Vitaterna MH Turek FW. Circadian Timing of Food Intake Contributes to Weight Gain. Obes (Silver Spring) (2009) 17:2100–2. 10.1038/oby.2009.264 PMC3499064 19730426

[34] Fonken LK Workman JL Walton JC Weil ZM Morris JS Haim A. Light at Night Increases Body Mass by Shifting the Time of Food Intake. Proc Natl Acad Sci USA (2010) 107:18664–9. 10.1073/pnas.1008734107 PMC2972983 20937863

[35] Scheer FA Hilton MF Mantzoros CS Shea SA. Adverse Metabolic and Cardiovascular Consequences of Circadian Misalignment. Proc Natl Acad Sci USA (2009) 106:4453–8. 10.1073/pnas.0808180106 PMC2657421 19255424

[36] Archer SN Laing EE Moller-Levet CS van der Veen DR Bucca G Lazar AS. Mistimed Sleep Disrupts Circadian Regulation of the Human Transcriptome. Proc Natl Acad Sci USA (2014) 111:E682–691. 10.1073/pnas.1316335111 PMC3926083 24449876

[37] Wefers J van Moorsel D Hansen J Connell NJ Havekes B Hoeks J. Circadian Misalignment Induces Fatty Acid Metabolism Gene Profiles and Compromises Insulin Sensitivity in Human Skeletal Muscle. Proc Natl Acad Sci USA (2018) 115:7789–94. 10.1073/pnas.1722295115 PMC6065021 29987027

[38] Bo S Fadda M Castiglione A Ciccone G De Francesco A Fedele D. Is the Timing of Caloric Intake Associated With Variation in Diet-Induced Thermogenesis and in the Metabolic Pattern? A Randomized Cross-Over Study. Int J Obes (Lond) (2015) 39:1689–95. 10.1038/ijo.2015.138 26219416

[39] Kessler K Hornemann S Petzke KJ Kemper M Kramer A Pfeiffer AF. The Effect of Diurnal Distribution of Carbohydrates and Fat on Glycaemic Control in Humans: A Randomized Controlled Trial. Sci Rep (2017) 7:44170. 10.1038/srep44170 28272464 PMC5341154

[40] Jakubowicz D Wainstein J Ahren B Bar-Dayan Y Landau Z Rabinovitz HR. High-Energy Breakfast With Low-Energy Dinner Decreases Overall Daily Hyperglycaemia in Type 2 Diabetic Patients: A Randomised Clinical Trial. Diabetologia (2015) 58:912–9. 10.1007/s00125-015-3524-9 25724569

[41] Lindgren O Mari A Deacon CF Carr RD Winzell MS Vikman J. Differential Islet and Incretin Hormone Responses in Morning Versus Afternoon After Standardized Meal in Healthy Men. J Clin Endocrinol Metab (2009) 94:2887–92. 10.1210/jc.2009-0366 19435824

[42] Gu C Brereton N Schweitzer A Cotter M Duan D Borsheim E. Metabolic Effects of Late Dinner in Healthy Volunteers-A Randomized Crossover Clinical Trial. J Clin Endocrinol Metab (2020) 105:2789–802. 10.1210/clinem/dgaa354 PMC7337187 32525525

[43] Martinez-Lozano N Tvarijonaviciute A Rios R Baron I Scheer F Garaulet M. Late Eating Is Associated With Obesity, Inflammatory Markers and Circadian-Related Disturbances in School-Aged Children. Nutrients (2020) 12:2881. 10.3390/nu12092881 PMC7551460 32967204

[44] Jakubowicz D Landau Z Tsameret S Wainstein J Raz I Ahren B. Reduction in Glycated Hemoglobin and Daily Insulin Dose Alongside Circadian Clock Upregulation in Patients With Type 2 Diabetes Consuming a Three-Meal Diet: A Randomized Clinical Trial. Diabetes Care (2019) 42:2171–80. 10.2337/dc19-1142 31548244

[45] Garaulet M Gomez-Abellan P Alburquerque-Bejar JJ Lee YC Ordovas JM Scheer FA. Timing of Food Intake Predicts Weight Loss Effectiveness. Int J Obes (Lond) (2013) 37:604–11. 10.1038/ijo.2012.229 PMC3756673 23357955

[46] Jakubowicz D Barnea M Wainstein J Froy O. High Caloric Intake at Breakfast vs. Dinner Differentially Influences Weight Loss of Overweight and Obese Women. Obes (Silver Spring) (2013) 21:2504–12. 10.1002/oby.20460 23512957

[47] Allison KC Goel N. Timing of Eating in Adults Across the Weight Spectrum: Metabolic Factors and Potential Circadian Mechanisms. Physiol Behav (2018) 192:158–66. 10.1016/j.physbeh.2018.02.047 PMC6019166 29486170

[48] Sandhu SK Tang TS. When’s Dinner? Does Timing of Dinner Affect the Cardiometabolic Risk Profiles of South-Asian Canadians at Risk for Diabetes. Diabetes Med (2017) 34:539–42. 10.1111/dme.13081 26802477

[49] Aljuraiban GS Chan Q Oude Griep LM Brown IJ Daviglus ML Stamler J. The Impact of Eating Frequency and Time of Intake on Nutrient Quality and Body Mass Index: The INTERMAP Study, a Population-Based Study. J Acad Nutr Diet (2015) 115:528–536 e521. 10.1016/j.jand.2014.11.017 25620753 PMC4380646

[50] Wehrens SMT Christou S Isherwood C Middleton B Gibbs MA Archer SN. Meal Timing Regulates the Human Circadian System. Curr Biol CB (2017) 27:1768–1775 e1763. 10.1016/j.cub.2017.04.059 28578930 PMC5483233

[51] LeCheminant JD Christenson E Bailey BW Tucker LA. Restricting Night-Time Eating Reduces Daily Energy Intake in Healthy Young Men: A Short-Term Cross-Over Study. Br J Nutr (2013) 110:2108–13. 10.1017/S0007114513001359 23702187

[52] Hibi M Masumoto A Naito Y Kiuchi K Yoshimoto Y Matsumoto M. Nighttime Snacking Reduces Whole Body Fat Oxidation and Increases LDL Cholesterol in Healthy Young Women. Am J Physiol Regulatory Integr Comp Physiol (2013) 304:R94–R101. 10.1152/ajpregu.00115.2012 23174861

[53] Bandin C Scheer FA Luque AJ Avila-Gandia V Zamora S Madrid JA. Meal Timing Affects Glucose Tolerance, Substrate Oxidation and Circadian-Related Variables: A Randomized, Crossover Trial. Int J Obes (Lond) (2015) 39:828–33. 10.1038/ijo.2014.182 25311083

[54] Qin LQ Li J Wang Y Wang J Xu JY Kaneko T. The Effects of Nocturnal Life on Endocrine Circadian Patterns in Healthy Adults. Life Sci (2003) 73:2467–75. 10.1016/S0024-3205(03)00628-3 12954455

[55] Almoosawi S Prynne CJ Hardy R Stephen AM. Diurnal Eating Rhythms: Association With Long-Term Development of Diabetes in the 1946 British Birth Cohort. Nutr Metab Cardiovasc Dis (2013) 23:1025–30. 10.1016/j.numecd.2013.01.003 23541169

[56] Almoosawi S Prynne CJ Hardy R Stephen AM. Time-Of-Day and Nutrient Composition of Eating Occasions: Prospective Association With the Metabolic Syndrome in the 1946 British Birth Cohort. Int J Obes (Lond) (2013) 37:725–31. 10.1038/ijo.2012.103 PMC3647231 22777542

[57] Kessler K Hornemann S Petzke KJ Kemper M Markova M Rudovich N. Diurnal Distribution of Carbohydrates and Fat Affects Substrate Oxidation and Adipokine Secretion in Humans. Am J Clin Nutr (2018) 108:1209–19. 10.1093/ajcn/nqy224 30541098

[58] Gupta NJ Kumar V Panda S. A Camera-Phone Based Study Reveals Erratic Eating Pattern and Disrupted Daily Eating-Fasting Cycle Among Adults in India. PloS One (2017) 12:e0172852. 10.1371/journal.pone.0172852 28264001 PMC5338776

[59] Roenneberg T Allebrandt KV Merrow M Vetter C. Social Jetlag and Obesity. Curr Biol CB (2012) 22:939–43. 10.1016/j.cub.2012.03.038 22578422

[60] Anton SD Moehl K Donahoo WT Marosi K Lee SA Mainous AG 3rd. Flipping the Metabolic Switch: Understanding and Applying the Health Benefits of Fasting. Obes (Silver Spring) (2018) 26:254–68. 10.1002/oby.22065 PMC5783752 29086496

[61] Chaix A Zarrinpar A Miu P Panda S. Time-Restricted Feeding Is a Preventative and Therapeutic Intervention Against Diverse Nutritional Challenges. Cell Metab (2014) 20:991–1005. 10.1016/j.cmet.2014.11.001 25470547 PMC4255155

[62] Antoni R Robertson T Robertson M Johnston J. A Pilot Feasibility Study Exploring the Effects of a Moderate Time-Restricted Feeding Intervention on Energy Intake, Adiposity and Metabolic Physiology in Free-Living Human Subjects. J Nutr Sci (2018) 7:e22. 10.1017/jns.2018.13

[63] Cai H Qin YL Shi ZY Chen JH Zeng MJ Zhou W. Effects of Alternate-Day Fasting on Body Weight and Dyslipidaemia in Patients With Non-Alcoholic Fatty Liver Disease: A Randomised Controlled Trial. BMC Gastroenterol (2019) 19:219. 10.1186/s12876-019-1132-8 31852444 PMC6921505

[64] Chow LS Manoogian ENC Alvear A Fleischer JG Thor H Dietsche K. Time-Restricted Eating Effects on Body Composition and Metabolic Measures in Humans Who are Overweight: A Feasibility Study. Obes (Silver Spring) (2020) 28:860–9. 10.1002/oby.22756 PMC7180107 32270927

[65] Cienfuegos S Gabel K Kalam F Ezpeleta M Wiseman E Pavlou V. Effects of 4- and 6-H Time-Restricted Feeding on Weight and Cardiometabolic Health: A Randomized Controlled Trial in Adults With Obesity. Cell Metab (2020) 32:366–78.e363. 10.1016/j.cmet.2020.06.018 32673591 PMC9407646

[66] Gabel K Hoddy KK Haggerty N Song J Kroeger CM Trepanowski JF. Effects of 8-Hour Time Restricted Feeding on Body Weight and Metabolic Disease Risk Factors in Obese Adults: A Pilot Study. Nutr Healthy Aging (2018) 4:345–53. 10.3233/NHA-170036 PMC6004924 29951594

[67] Gabel K Marcell J Cares K Kalam F Cienfuegos S Ezpeleta M. Effect of Time Restricted Feeding on the Gut Microbiome in Adults With Obesity: A Pilot Study. Nutr Health (2020) 26:79–85. 10.1177/0260106020910907 32228124

[68] Hutchison AT Regmi P Manoogian ENC Fleischer JG Wittert GA Panda S. Time-Restricted Feeding Improves Glucose Tolerance in Men at Risk for Type 2 Diabetes: A Randomized Crossover Trial. Obes (Silver Spring) (2019) 27:724–32. 10.1002/oby.22449 31002478

[69] Jamshed H Beyl RA Della Manna DL Yang ES Ravussin E Peterson CM. Early Time-Restricted Feeding Improves 24-Hour Glucose Levels and Affects Markers of the Circadian Clock, Aging, and Autophagy in Humans. Nutrients (2019) 11:1234. 10.3390/nu11061234 PMC6627766 31151228

[70] Ravussin E Beyl RA Poggiogalle E Hsia DS Peterson CM. Early Time-Restricted Feeding Reduces Appetite and Increases Fat Oxidation But Does Not Affect Energy Expenditure in Humans. Obes (Silver Spring) (2019) 27:1244–54. 10.1002/oby.22518 PMC6658129 31339000

[71] Jones R Pabla P Mallinson J Nixon A Taylor T Bennett A. Two Weeks of Early Time-Restricted Feeding (eTRF) Improves Skeletal Muscle Insulin and Anabolic Sensitivity in Healthy Men. Am J Clin Nutr (2020) 112:1015–28. 10.1093/ajcn/nqaa192 PMC7528549 32729615

[72] Karras SN Koufakis T Adamidou L Antonopoulou V Karalazou P Thisiadou K. Effects of Orthodox Religious Fasting Versus Combined Energy and Time Restricted Eating on Body Weight, Lipid Concentrations and Glycaemic Profile. Int J Food Sci Nutr (2021) 72:82–92. 10.1080/09637486.2020.1760218 32362210

[73] Kesztyus D Cermak P Gulich M Kesztyus T. Adherence to Time-Restricted Feeding and Impact on Abdominal Obesity in Primary Care Patients: Results of a Pilot Study in a Pre-Post Design. Nutrients (2019) 11:2854. 10.3390/nu11122854 PMC6950236 31766465

[74] Li C Xing C Zhang J Zhao H Shi W He B. Eight-Hour Time-Restricted Feeding Improves Endocrine and Metabolic Profiles in Women With Anovulatory Polycystic Ovary Syndrome. J Trans Med (2021) 19:148. 10.1186/s12967-021-02817-2 PMC8045367 33849562

[75] Lowe DA Wu N Rohdin-Bibby L Moore AH Kelly N Liu YE. Effects of Time-Restricted Eating on Weight Loss and Other Metabolic Parameters in Women and Men With Overweight and Obesity: The TREAT Randomized Clinical Trial. JAMA Intern Med (2020) 180:1491–9. 10.1001/jamainternmed.2020.4153 PMC7522780 32986097

[76] Martens CR Rossman MJ Mazzo MR Jankowski LR Nagy EE Denman BA. Short-Term Time-Restricted Feeding Is Safe and Feasible in Non-Obese Healthy Midlife and Older Adults. Geroscience (2020) 42:667–86. 10.1007/s11357-020-00156-6 PMC7206473 31975053

[77] McAllister MJ Pigg BL Renteria LI Waldman HS. Time-Restricted Feeding Improves Markers of Cardiometabolic Health in Physically Active College-Age Men: A 4-Week Randomized Pre-Post Pilot Study. Nutr Res (2020) 75:32–43. 10.1016/j.nutres.2019.12.001 31955013

[78] Moro T Tinsley G Bianco A Marcolin G Pacelli QF Battaglia G. Effects of Eight Weeks of Time-Restricted Feeding (16/8) on Basal Metabolism, Maximal Strength, Body Composition, Inflammation, and Cardiovascular Risk Factors in Resistance-Trained Males. J Trans Med (2016) 14:290. 10.1186/s12967-016-1044-0 PMC5064803 27737674

[79] Parr EB Devlin BL Lim KHC Moresi LNZ Geils C Brennan L. Time-Restricted Eating as a Nutrition Strategy for Individuals With Type 2 Diabetes: A Feasibility Study. Nutrients (2020) 12:3228. 10.3390/nu12113228 PMC7690416 33105701

[80] Parr EB Devlin BL Radford BE Hawley JA. A Delayed Morning and Earlier Evening Time-Restricted Feeding Protocol for Improving Glycemic Control and Dietary Adherence in Men With Overweight/Obesity: A Randomized Controlled Trial. Nutrients (2020) 12:505. 10.3390/nu12020505 PMC7071240 32079327

[81] Lundell LS Parr EB Devlin BL Ingerslev LR Altintas A Sato S. Time-Restricted Feeding Alters Lipid and Amino Acid Metabolite Rhythmicity Without Perturbing Clock Gene Expression. Nat Commun (2020) 11:4643. 10.1038/s41467-020-18412-w 32938935 PMC7495469

[82] Peeke PM Greenway FL Billes SK Zhang D Fujioka K. Effect of Time Restricted Eating on Body Weight and Fasting Glucose in Participants With Obesity: Results of a Randomized, Controlled, Virtual Clinical Trial. Nutr Diabetes (2021) 11:6. 10.1038/s41387-021-00149-0 33446635 PMC7809455

[83] Phillips NE Mareschal J Schwab N Manoogian ENC Borloz S Ostinelli G. The Effects of Time-Restricted Eating Versus Standard Dietary Advice on Weight, Metabolic Health and the Consumption of Processed Food: A Pragmatic Randomised Controlled Trial in Community-Based Adults. Nutrients (2021) 13:1042. 10.3390/nu13031042 33807102 PMC8004978

[84] Sutton EF Beyl R Early KS Cefalu WT Ravussin E Peterson CM. Early Time-Restricted Feeding Improves Insulin Sensitivity, Blood Pressure, and Oxidative Stress Even Without Weight Loss in Men With Prediabetes. Cell Metab (2018) 27:1212–21.e1213. 10.1016/j.cmet.2018.04.010 29754952 PMC5990470

[85] Tinsley GM Forsse JS Butler NK Paoli A Bane AA La Bounty PM. Time-Restricted Feeding in Young Men Performing Resistance Training: A Randomized Controlled Trial. Eur J Sport Sci (2017) 17:200–7. 10.1080/17461391.2016.1223173 27550719

[86] Wilkinson MJ Manoogian ENC Zadourian A Lo H Fakhouri S Shoghi A. Ten-Hour Time-Restricted Eating Reduces Weight, Blood Pressure, and Atherogenic Lipids in Patients With Metabolic Syndrome. Cell Metab (2020) 31:92–104 e105. 10.1016/j.cmet.2019.11.004 31813824 PMC6953486

[87] Zeb F Wu X Chen L Fatima S Haq IU Chen A. Effect of Time-Restricted Feeding on Metabolic Risk and Circadian Rhythm Associated With Gut Microbiome in Healthy Males. Br J Nutr (2020) 123:1216–26. 10.1017/S0007114519003428 31902372

[88] Kesztyus D Fuchs M Cermak P Kesztyus T. Associations of Time-Restricted Eating With Health-Related Quality of Life and Sleep in Adults: A Secondary Analysis of Two Pre-Post Pilot Studies. BMC Nutr (2020) 6:76. 10.1186/s40795-020-00402-2 33327959 PMC7745395

[89] Poggiogalle E Jamshed H Peterson CM. Circadian Regulation of Glucose, Lipid, and Energy Metabolism in Humans. Metabolism: Clin Exp (2018) 84:11–27. 10.1016/j.metabol.2017.11.017 PMC5995632 29195759

[90] Middleton KR Anton SD Perri MG. Long-Term Adherence to Health Behavior Change. Am J Lifestyle Med (2013) 7:395–404. 10.1177/1559827613488867 27547170 PMC4988401

[91] Kanerva N Kronholm E Partonen T Ovaskainen ML Kaartinen NE Konttinen H. Tendency Toward Eveningness Is Associated With Unhealthy Dietary Habits. Chronobiol Int (2012) 29:920–7. 10.3109/07420528.2012.699128 22823875

[92] Munoz JSG Canavate R Hernandez CM Cara-Salmeron V Morante JJH. The Association Among Chronotype, Timing of Food Intake and Food Preferences Depends on Body Mass Status. Eur J Clin Nutr (2017) 71:736–42. 10.1038/ejcn.2016.182 27650874

[93] Liu H Javaheri A Godar RJ Murphy J Ma X Rohatgi N. Intermittent Fasting Preserves Beta-Cell Mass in Obesity-Induced Diabetes via the Autophagy-Lysosome Pathway. Autophagy (2017) 13:1952–68. 10.1080/15548627.2017.1368596 PMC5788488 28853981

[94] Sato M Murakami M Node K Matsumura R Akashi M. The Role of the Endocrine System in Feeding-Induced Tissue-Specific Circadian Entrainment. Cell Rep (2014) 8:393–401. 10.1016/j.celrep.2014.06.015 25017062

[95] Landgraf D Tsang AH Leliavski A Koch CE Barclay JL Drucker DJ. Oxyntomodulin Regulates Resetting of the Liver Circadian Clock by Food. Elife (2015) 4:e06253. 10.7554/eLife.06253 25821984 PMC4426666

[96] Chaudhari A Gupta R Makwana K Kondratov R. Circadian Clocks, Diets and Aging. Nutr Healthy Aging (2017) 4:101–12. 10.3233/NHA-160006 PMC5389023 28447065

[97] Jaspers RT Zillikens MC Friesema EC delli Paoli G Bloch W Uitterlinden AG. Exercise, Fasting, and Mimetics: Toward Beneficial Combinations? FASEB J Off Publ Fed Am Societies Exp Biol (2017) 31:14–28. 10.1096/fj.201600652r 27729415

